# Isolation of epithelial cells from acrylic removable dentures and gender identification by amplification of SRY gene using real time PCR

**DOI:** 10.4103/0974-2948.71055

**Published:** 2010

**Authors:** Renjith George, G Sriram, TR Saraswathi, B Sivapathasundharam

**Affiliations:** *Department of Oral Pathology, Meenakshi Ammal Dental College, Chennai, India*

**Keywords:** Acrylic removable dentures, gender identification, real-time PCR, SRY

## Abstract

This study evaluates the usefulness of acrylic dentures as the source of DNA for forensic analysis. Thirty-eight samples (21 males and 17 females) were collected and stored for different time periods. The epithelial cells adhered to the dentures were retrieved and the genomic DNA was extracted. All the samples yielded sufficient amount of DNA for analysis irrespective of the storage time. Gender determination was done by amplification of the sex determining region on the Y chromosome (SRY) using real-time polymerase chain reaction with 100% accuracy, within minimal time. With this study, we conclude that saliva-stained acrylic dentures can act as a source of forensic DNA and co-amplification of SRY gene with other routine sex typing markers will give unambiguous gender identification.

## Introduction

Dental evidence derived from the history of dental treatment is one of the most reliable methods for personal identification of unknown bodies. The development of DNA studies has provided another powerful approach for the identification of unknown remains and other biological evidence in the field of forensic medicine. The usual source of DNA from the oral and maxillofacial region is pulpal tissue; but saliva-stained stamps, human skin, cigarette butts, receptacle for drinks, tooth brushes, chewed gums, bitten food stuff, impressions made for dental treatment, used dental floss, histopathologic sections made from biopsies, cytologic smears, and oral prostheses can also be used as sources of DNA as the saliva contains many epithelial cells.[1]

Dental prostheses in the oral cavity are exposed to saliva and when they are removed, salivary components may remain on the surface. Because submandibular sublingual saliva promotes the adhesion of microorganisms to polymethyl methacrylate,[1] it may also mediate the adhesion of oral epithelial cells and leukocytes to the resin prostheses. Based on this, we hypothesize that epithelial cells would be present adhering to the resin prostheses and DNA could be extracted from the epithelial cells. In the present study, we isolated epithelial cells from resin prostheses and analyzed the DNA for the sex determining region on the Y chromosome (SRY) and its use in determining the sex of the individual.

## Materials and Methods

The present study was carried out in the Department of Oral and Maxillofacial Pathology and Department of Prosthodontics, Meenakshi Ammal Dental College, Chennai, and in Shrimpex Biotech Lab, Chennai. Acrylic removable partial dentures of 38 denture wearers were duplicated and the subjects were instructed to use it for one week and to return it without washing. The samples were unwashed and kept at room temperature, in sterile closed containers. Care was taken to avoid any kind of contamination. To assess the possibility of any genetic abnormality and chimerism, information regarding any diagnosed syndromes, family history, bone marrow transplantation, and family details of female subjects were also recorded through personal interview using a questionnaire.

Eighteen samples were subjected to analysis immediately with no storage time, 10 after 1 month and 10 after 2 months of storage time. Period of time they had been used in the oral cavity was also recorded. After the storage period, the acrylic dentures were washed in saline and shavings from the tissue-contacting surface were taken. The wash saline and shavings were collected in a sterile plastic container. Care was taken to avoid contamination. The wash saline was centrifuged at 13,000 rpm for 1 min and the supernatant was discarded. The sediment was transferred to sterile 1.5 mL Eppendorf collection tube.

### DNA extraction and quantification

DNA extraction was performed using Real Genomics YGB 100 (Real Biotech Corporation, Taiwan) DNA extraction kit. Quantification of DNA extracted from each sample was performed using Nanodrop ND-1000 spectrophotometer. DNA in the solution was quantified by the absorbance of light (260 nm) in spectrophotometer.

Concentration of DNA (ng/µL)=A260 × conversion factor (OD unit=50 ng/µL) × dilution factor.

### Amplification and detection of SRY gene

The various primers and probes used for the amplification and detection of *SRY* gene using *Taq* PCR master mix (Qiagen, India) and real-time polymerase chain reaction (PCR) are given in Table 1. The PCR cycle consisted of initial denaturation at 95°C for 7 min, followed by 40 cycles of annealing at 95°C for 15 s, and extension at 60°C for 1 min. The presence of target gene *SRY* is automatically detected by the Realplex Master Cycler (Eppendorff, Germany) and the result is plotted in the form of a visualization chart with red color indicating positive and green color indicating negative.

**Table 1:** Primers and probe for amplification and detection of SRY gene

Primers & probe	Sequence
Forward primer	GCG ACC CAT GAA CGC ATT
Reverse primer	AGT TTC GCA TTC TGG GAT TCT CT
Probe	FAM- TGG TCT CGC GAT CAG AGG CGC- TAMR

## Results

The amount and purity of DNA extracted from each sample was performed using Nanodrop ND 1000 Spectrophotometer. The average yield of DNA from the collected samples (without considering the surface area of the denture) was 34.60 ng/μL. The average yield of DNA from various samples based on the storage time and sex are presented in Table 2. Statistical analysis showed that the storage time had no correlation on the DNA yield.

**Table 2:** Average yields of DNA from various samples based on storage time and sex

Subjects	Number	DNA yield at different storage time(ng/*μ*l)		
		0 days (Mean±SD)	30 days (Mean±SD)	60 days (Mean±SD)
All Subjects	38	30.91±20.60	26.68±22.81	49.19±35.50
Males	21	16.95±10.22	33.10±24.85	57.72±21.81
Females	17	44.87±18.95	11.69±2.45	40.65±46.67

The purity of the DNA extracted was expressed as the ratio of absorption at 260 nm/280 nm. DNA has peak absorption at 280 nm, protein at 230 nm, and RNA at 260 nm. The ratio of absorption at 260 nm/280 nm of more than 1.9 indicates RNA contamination and a ratio less than 1.7 indicates protein contamination, whereas pure DNA has a ratio of 1.8.[2] The average purity of DNA in the present study was 2.11, indicating RNA contamination; however, the purity varied from sample to sample. The mean purity of DNA of samples with different storage times are presented in Table 3. Statistical analysis showed that the storage time had no correlation on the purity of the DNA.

**Table 3:** Mean purity of DNA of samples with different storage times

Subjects	Number	Purity of DNA (expressed as ratio of absorption at 260 nm/ 280 nm)		
		0 days (Mean±SD)	30 days (Mean±SD)	60 days (Mean±SD)
All Subjects	38	2.28±1.36	1.85±0.24	2.11±0.73
Males	21	2.81±1.74	1.90±0.26	2.07±0.10
Females	17	1.74±0.51	1.70±0.06	2.14±1.08

The actual sex and the sex determined based on the presence or absence of *SRY* gene is presented in Table 4. All the subjects were identified with 100% accuracy as the appropriate gender based on the presence or absence of *SRY* gene. Analysis of the family history of the 17 female subjects revealed that 8 of them had at least 1 son. Of the 8 female subjects with at least 1 son, none showed a false-positive result for *SRY*.

**Table 4:** The actual sex and the sex determined based on the presence or absence of SRY gene

Sample number	Actual gender	Gender based on SRY gene
1.	Male	Male
2.	Male	Male
3.	Male	Male
4.	Male	Male
5.	Female*	Female
6.	Male	Male
7.	Male	Male
8.	Male	Male
9.	Female*	Female
10.	Male	Male
11.	Male	Male
12.	Male	Male
13.	Male	Male
14.	Female	Female
15.	Female	Female
16.	Female*	Female
17.	Male	Male
18.	Male	Male
19.	Male	Male
20.	Male	Male
21.	Male	Male
22.	Female	Female
23.	Male	Male
24.	Male	Male
25.	Male	Male
26.	Male	Male
27.	Female*	Female
28.	Female*	Female
29.	Female*	Female
30.	Female	Female
31.	Male	Male
32.	Female	Female
33.	Female	Female
34.	Female	Female
35.	Female	Female
36.	Female*	Female
37.	Female*	Female
38.	Female	Female

* indicates females with at least one son

## Discussion

Various methods are employed in forensic dentistry for gender identification, which includes morphology of skull and mandible, metric features, and DNA analysis.[3] Visual methods are reliable but are subject to interobserver variation and lack accuracy. Microscopic methods, such as identifying gender by Barr bodies show diminishing accuracy with the time elapsed after death.[4] On the other hand, gender identification using advanced methods are found to be more accurate with reproducible results.

**Figure 1:**
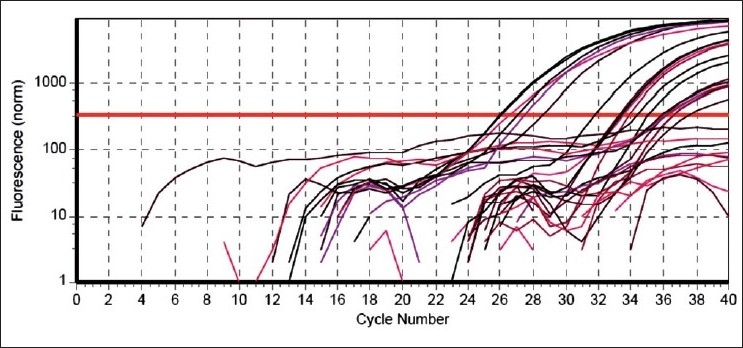
Real-time PCR amplification plot for SRY gene. The horizontal red line indicates the threshold value of fluorescence. All the samples positive for *SRY* gene show amplification of the target sequence that attains the threshold fluorescence at varying cycles of amplification. Those samples negative for the *SRY* gene show the fluorescence to be below the threshold value (red line)

There are many potential sources from which DNA can be extracted, but the amount of DNA yield is affected by various factors. Inoue *et al*.[1] reported a DNA yield of 35.7 ng to 1.52 μg/μL from acrylic resin blocks prepared from dentures. Tanaka *et al*.[5] wereable to retrieve 10 430 ng/μL of DNA from tooth brushes used by their subjects. Hochmeister *et al*.[6] obtained a range from 2 to 160 ng/μL of DNA from cigarette butt samples. Sinclair *et al*.[7] reported the yield of DNA from saliva-stained stamps ranging from 5 to 50 ng/μL and 1 to 30 ng/μL from the flap of the envelope. Lorente *et al*.[8] retrieved more than 30 ng of DNA from as little as 1.5 mg of dandruff. In the present study, irrespective of the storage time and period of use in the oral cavity, the DNA yield from 38 samples varied between 2.26L and 116.92 ng/μL. However, the amount of DNA yield as measured by spectrophotometry in most of the studies reported, including the present one, is not indicative of human DNA alone. It also includes the DNA from oral microflora, as purification of the DNA is not performed and is not necessary for PCR. Even the minute traces of human DNA are sufficient to amplify a specific gene target using PCR. Real-time PCR offers an added advantage over the conventional PCR in that the amplification of the specified gene target is shown as a graph representing the amount of fluorescence emitted by PCR products after each cycle [Figure 1].

DNA-based gender determination can be performed using various sex typing markers, such as amelogenin, centromeric alphoid repeats, ZFX/ZFY (zinc finger genes), and the *SRY* gene.[9] In humans, where there are distinct sex chromosomes, the X and the Y chromosomes, it is the presence of the Y chromosome that specifies the male development. More specifically, there is a gene on the Y chromosome called the sex determining region of the Y chromosome (*SRY*) that causes the development of male characters.[7] Hence the detection of *SRY* amplicons, would distinguish an authentic male DNA sample from a female DNA sample, as a female DNA lacks the presence of the *SRY* gene.[10,11]

*SRY* gene is present on the Y chromosome, and it binds to other DNA molecules and in doing so distorts the Y chromosome dramatically out of shape. This alters the properties of the DNA and likely alters the expression of a number of genes, leading to testis formation. Most XX men who lack a Y chromosome do still have a copy of the *SRY* region on one of their X chromosomes. This copy accounts for their maleness. However, because the remainder of the Y chromosome is missing, they frequently do not develop secondary sexual characteristics in the usual way.[10]

The detection of *SRY* gene in the DNA from a forensic sample can be confirmatory to type the gender as male. Kastelic *et al*.[12] reported gender identification with 100% accuracy using the *SRY* gene. In the present study, we were able to determine the gender of all the 38 samples by detecting the *SRY* region with 100% accuracy.

However, gender identification based on the presence or absence of a single gene cannot be considered as confirmatory, as phenotype-based legal gender may sometimes be contradictory to the genetic sex in conditions such as genetic diversity, intersex conditions, and transsexualism. Many investigators suggested co-amplification of *SRY* gene along with amelogenin for unambiguous gender identification.[11,13–15] Turner syndrome (46,X0), Klinefelter syndrome (46,XXY), androgen insensitivity syndrome, and 47,XYY syndrome may show a discrepancy between the genotype and the phenotype of a person.[16] Dauwerse *et al*.[17] reported a case of an infertile male with normal development of male genitalia having 46,XX karyotype, but *SRY* gene was identified to be inserted onto the terminal end of chromosome 16.

In case of chimerism (donor alleles in recipient DNA in case of bone marrow transplant) or microchimerism (occurrence of cell-free DNA in the maternal serum), samples, such as blood and buccal swab may show erroneous results in sex typing with the *SRY* gene.[18] Chimeras express or more genetically distinct cell types. Nicole *et al*.[19] reported that sex determination can be erroneous in samples taken from bone marrow-transplanted subjects or from women carrying a male fetus. In individuals with a bone marrow transplant, the patient’s bone marrow is destroyed and replaced by healthy marrow or stem cells derived from the donor’s marrow. Hence these blood cells would be expressing a genetic pattern of the donor. So, the forensically used short tandem repeats found in the recipient’s blood would be identical to that of the donor.[19] Dauber *et al*.[20] detected alleles of the donor in DNA from buccal swabs or fingernails from the patients even 5 years after transplantation. In case of difference in the sex between the donor and the recipient, PCR-based gender identification can lead to wrong conclusions.[20] According to Cohena *et al*.,[21] if umbilical cord blood can be used as an alternative source of hematopoietic progenitors for allogeneic stem cell transplantation, forensic consequences as in case of gender determination can be eliminated.

Similarly, the Y-specific alleles of the male fetus can be detected in the maternal blood during pregnancy (microchimerism). Bianchi[22] stated that sex determination can be erroneous when investigating biological blood traces from women who are pregnant with male fetuses. Peripheral blood microchimerism after pregnancy or solid organ transplantation may affect the forensic sex typing, while using the blood as the sample.[23–25] Fetal cells can migrate into the female body tissues, such as skin, blood, muscle, and solid organs, during pregnancy and can persist for decades (persisting fetal microchimerism).[26] In the present study, none of the samples from the female subjects with atleast 1 son, showed amplification of the *SRY* gene. Hence there was no evidence of chimerism detectable in the females included in the present study.

Such variation in the phenotypic and genotypic sex is not limited only to the presence of the *SRY* gene. The most commonly used gene for gender determination is amelogenin (AMEL) gene. Ajay *et al*.[27] have reported the use of AMEL gene in gender determination from DNA extracted from dental pulp. Similar to the *SRY* gene, reliability of sex determination using AMEL gene-specific fragments has been questioned. Santos *et al*.,[19] Thangaraj *et al*.,[13] Steinlechner *et al*.,[15] Chang *et al*.[28] and Kao *et al*.[29] found a deletion of the amelogenin gene on the Y chromosome, which led to the disappearance of the usually longer Y-specific fragment, and therefore to the detection of only the smaller X-specific signal, generating an apparently female genotype. Kashyap *et al*.[30] reported 0.23% failure rate in amelogenin-based gender typing among Indian population. According to their findings, they recommended, that a Y-specific locus should routinely be included along with an X-specific marker in forensic sex tests to avoid false statements on gender determination.

## Conclusion

In the present study, we were able to successfully extract the DNA from exfoliated epithelial cells retained in the dental prostheses made of acrylic resin used by the subjects irrespective of the storage time and type the gender using real-time PCR-based amplification of male-specific marker *SRY* gene with 100% accuracy. None of the female samples showed amplification for *SRY* gene, and there was no evidence of chimerism that could lead to false gender identification. However, the findings of the present study are limited to normal individuals. The accuracy and specificity of the *SRY* in gender identification needs to be confirmed by extending the study in various syndromic conditions that could alter the expression of *SRY*.
